# Clinical benefits of incorporating doxycycline into a canine heartworm treatment protocol

**DOI:** 10.1186/s13071-017-2446-4

**Published:** 2017-11-09

**Authors:** C. Thomas Nelson, Elizabeth S. Myrick, Thomas A. Nelson

**Affiliations:** 1 Animal Medical Centers of NE Alabama, 719 Quintard Ave, Anniston, AL 36201-5757 USA; 2 Banfield Pet Hospital, Birmingham, AL USA; 3 BluePearl Veterinary Partners, Lawrenceville, Georgia USA

**Keywords:** Heartworm, Canine, Melarsomine, Doxycycline, Ivermectin, Prednisone, Respiratory complications, Mortality, Treatment protocol

## Abstract

**Background:**

The objective of heartworm treatment is to improve the clinical condition of the patient and to eliminate pre-cardiac, juvenile, and adult worm stages with minimal complications. Pulmonary thromboembolisms are an inevitable consequence of worm death and can result in severe pulmonary reactions and even death of the patient. To minimize these reactions, various treatment protocols involving melarsomine, the only adulticidal drug approved by the US Food and Drug Administrations (FDA), in conjunction with macrocyclic lactone heartworm preventives and glucocorticosteroids have been advocated. The discovery of the bacterial endosymbiont *Wolbachia* in *Dirofilaria immitis* has led to several experimental studies examining the effects of administering doxycycline to reduce or eliminate *Wolbachia* organism. These studies have shown a decrease in gross and microscopic pathology of pulmonary parenchyma in experimental heartworm infections pretreated with doxycycline before melarsomine administration.

**Methods:**

Electronic medical records from a large veterinary practice in northeast Alabama were searched to identify dogs treated for heartworms with melarsomine from January 2005 through December 2012. The search was refined further to select for dogs that met the following criteria: 1) received two or three doses of ivermectin heartworm preventive prior to melarsomine injections, 2) received one injection of melarsomine followed by two injections 4 to 8 weeks later, and 3) were treated with prednisone following melarsomine injections. The dogs were then divided into those that also were treated with doxycycline 10 mg/kg BID for 4 weeks (Group A, *n* = 47) and those that did not receive doxycycline (Group B, n = 47). The medical notes of all 94 cases were then reviewed for comments concerning coughing, dyspnea, or hemoptysis in the history, physical exam template, or from telephone conversations with clients the week following each visit. Any dog that died within one year of treatment from either cardiovascular or pulmonary problems was noted.

**Results:**

Dogs from Group A receiving doxycycline had fewer respiratory complications (6.52%) and heartworm disease-related deaths (0%) than Group B (19.14% and 4.25%, respectively).

**Conclusions:**

Although there are not enough cases to indicate statistical significance, the results strongly suggest that including doxycycline into canine heartworm treatment protocols decreases post-treatment complications and mortality in naturally infected clinical cases.

## Background

A thorough understanding of the heartworm life cycle, effects of macrocyclic lactones and melarsomine on various heartworm life stages, and the host–parasite relationship is critical in managing heartworm disease and in development of effective treatment protocols. A synopsis of this information compiled from decades of research can be found in the American Heartworm Society’s Guidelines [1]. These guidelines outline important principles of treatment, such as the fact that activity level of the dog is the single most important factor determining severity of disease [2, 3]. The guidelines also discuss that two injections of melarsomine kill 90% of the adult worms, the three-injection protocol kills 98% of the adult worms [4], and prednisone is the treatment of choice for the inflammation associated with pulmonary thromboembolisms [5, 6].

The discovery of the bacterial endosymbiont *Wolbachia* in *Dirofilaria immitis* [7] and evidence that this organism plays a role in the pathogenesis of heartworm disease [8–11] has led to several experimental studies examining the effects of administering doxycycline to reduce or eliminate the *Wolbachia* organism from *D. immitis*. These studies have shown a decrease in gross and microscopic pathology of pulmonary parenchyma in experimental heartworm infections pretreated with doxycycline before melarsomine administration [12, 13]. Doxycycline administered at 10 mg/kg twice daily (BID) for 4 weeks has been shown to reduce or eliminate the *Wolbachia* organisms for up to 12 months [14]. Additional studies have shown that administration of doxycycline in combination with ivermectin provided more rapid adulticidal activity than ivermectin alone [15] as well as more effectively reducing *Wolbachia* numbers than doxycycline alone [16]. Furthermore, dogs treated with doxycycline and ivermectin prior to receiving melarsomine had less severe arterial lesions and virtual absence of thrombi [13].

This wealth of information has led to the widespread use of doxycycline by veterinary practitioners in heartworm treatment protocols. If used, doxycycline should be administered before administration of melarsomine so the *Wolbachia* organisms and *Wolbachia*-associated molecules are reduced or absent when the worms die and fragment. The multi-modal treatment protocol shown in Table 1 was developed by the lead author based on published research and clinical experience and has been in use since 2005. This protocol is in widespread use and has been included in multiple publications [1, 17, 18].

**Table 1:** Protocol developed and adopted by Animal Medical Centers of Northeast Alabama for heartworm treatment

Day	Treatment
0	Dog is diagnosed and verified as heartworm positive. If the dog is symptomatic, attempt to stabilize the dog with ACE inhibitors, furosemide, and if severely ascitic, abdominal drainage. Prednisone at 0.5 mg/kg twice daily 1st week, 0.5 mg/kg once daily 2nd week, 0.5 mg/kg every other day 3rd & 4th week. Administer ivermectin heartworm preventive (If microfilariae are present, pre-treat with antihistamine and glucocorticosteroids if not already on prednisone.) Begin exercise restriction. The more pronounced the symptoms, the stricter the exercise restriction.
1–28	Doxycycline 10 mg/kg BID for 4 weeks.
30	Administer ivermectin heartworm preventive.
60	Administer ivermectin heartworm preventive. First melarsomine injection 2.5 mg/kg IM (Day 61). Prescribe prednisone 0.5 mg/kg twice daily 1st week, 0.5 mg/kg once daily 2nd week, 0.5 mg/kg every other day 3rd & 4th week. Decrease activity level even further. Cage rest in more severe cases.
90	Administer ivermectin heartworm preventive. Second and third melarsomine injections 2.5 mg/kg IM (Day 90 & 91). Prescribe prednisone 0.5 mg/kg twice daily 1st week, 0.5 mg/kg once daily 2nd week, 0.5 mg/kg every other day 3rd & 4th week. Continue exercise restriction for 6 to 8 weeks after last melarsomine injections. Antigen test 6 months after completion.

*ACE* angiotensin converting enzyme inhibitor

While there is an abundance of data on the use of doxycycline in treating experimentally infected dogs in laboratory settings, the clinical benefits in naturally infected client-owned dogs have been largely anecdotal. In an attempt to document the clinical benefits of adding doxycycline to heartworm treatment protocols in naturally infected client-owned dogs, a comprehensive independent records review was performed at a large multi-doctor practice in northeast Alabama.

## Method

Electronic medical records were searched to identify all dogs treated for heartworms with melarsomine from January 2005 through December 2012. The search was refined further to select for dogs that met the following criteria: 1) received two or three doses of an ivermectin-based heartworm preventive at standard preventive doses prior to melarsomine injections, 2) received one injection of melarsomine, 2.5 mg/kg, followed by two injections at the same dose 4 to 8 weeks later, and 3) were treated with prednisone following melarsomine injections as indicated in Table 1. The dogs were then divided into those that also were treated with doxycycline at 10 mg/kg BID for 4 weeks (Group A, *n* = 47) and those that did not receive doxycycline (Group B, n = 47). No effort was made to divide the animals into classes as all dogs were treated with the three-dose protocol recommended for Class 3 heartworm disease.

The medical notes of all 94 cases were independently reviewed for comments concerning coughing, dyspnea, or hemoptysis in the history, physical exam template, or from telephone conversations with clients the week following each visit. Results were recorded as either having clinically evident respiratory complications or none. Any dog that died within one year of treatment from either cardiovascular or pulmonary problems was also noted. One dog from Group A that was hit by a car and died 8 months after treatment was excluded, lowering Group A to *n* = 46.

## Results

Three of 46 dogs (6.52%) in Group A were reported to be coughing, dyspneic, or exhibiting hemoptysis. There were no deaths in Group A, which contained the dogs receiving doxycycline. In Group B, 9 of 47 dogs (19.14%) were reported to have coughing, dyspnea, or hemoptysis, and 2 of 47 (4.25%) died as a result of cardiovascular or pulmonary problems attributed to heartworm disease (Table 2). Statistical analysis by chi square for respiratory signs indicated a trend towards significance with a *p*-value of 0.069.

**Table 2:** Results of records review

	Group A	Group B
Protocol	Ivm/Doxy/Mel/Pred	Ivm/Mel/Pred
Number in group	*N* = 46	*N* = 47
Post-treatment respiratory signs	3 (6.52%)	9 (19.14%)
Mortality	0 (0%)	2 (4.25%)

*Ivm* ivermectin, *Doxy* doxycycline, *Mel* melarsomine, *Pred* prednisone

## Discussion

Clinical field trials using melarsomine alone reported an adverse reaction rate of coughing in 22.2% of dogs, dyspnea in 2.6%, and hemoptysis in 1.6% [19]. Although all three pulmonary reactions were combined for the two groups in the present study, one would expect the dyspnea and hemoptysis patients also to be coughing. If this assumption is correct, the percentages of pulmonary reactions are very similar between Group B (19.14%) and those reported in the clinical field trials (22.2%). In the clinical field trials, a mortality rate of 5.2% for Class 1 and 2 and 18.2% for Class 3 due to the disease or treatment-related death was reported. The combined mortality rate for all classes was 7.0% in clinical field trials, which is noticeably higher than the 4.25% reported in Group B. All dogs in Group B, however, were treated with the three-dose protocol as recommended by the American Heartworm Society [1] because of the increased safety and efficacy and also received prednisone as described in Table 1. This likely contributed to the overall lower mortality rate in Group B as compared with clinical field trials.

Dogs in Group A (included doxycycline) had 12.62% fewer pulmonary reactions (19.14% –6.52%), which is 65% fewer (12.62% ÷ 19.14%) than Group B. There was also 0% mortality in Group A. One could argue that the dogs in Group A may not have been as severely infected and/or had less pulmonary disease than Group B. While no attempt was made to stage the severity of disease, during the years 2005–2007 doxycycline was only used in the more advanced Class 3 cases of heartworm disease.

## Conclusions

Although there are not enough cases to indicate statistical significance, the results strongly suggest that including doxycycline into canine heartworm treatment protocols decreases post-treatment complications and mortality in naturally infected clinical cases. This is consistent with what has been published in multiple studies utilizing experimental infections and anecdotal reports.
